# Sleep apnea–hypopnea syndrome caused by ankylosing spondylitis: A case report

**DOI:** 10.1097/MD.0000000000020055

**Published:** 2020-05-08

**Authors:** Yan Wang, Shan Lin, Chenxi Li, Yingqing Shi, Wei Guan

**Affiliations:** a Department of Respiratory Medicine, Qinghai University Affiliated Hospital, Xining; b Department of Medical Intensive Care Unit, The First Affiliated Hospital of Sun Yat-Sen University; c Department of Respiratory and Critical Medicine, Baoan Central Hospital of Shenzhen and The Fifth Affiliated Hospital of Shenzhen University, Shenzhen, Guangdong, China.

**Keywords:** ankylosing spondylitis, autoimmune disease, case report, sleep apnea–hypopnea syndrome, TNF-α

## Abstract

**Introduction:**

Sleep apnea–hypopnea syndrome (SAHS) is a multifactorial disease characterized by recurrent hypopnea or respiratory interruption during sleep, which causes intermittent hypoxemia, hypercapnia, and sleep structure disturbances. An association between ankylosing spondylitis (AS) and the type of SAHS has rarely been reported in the literature. Here, we present a case of SAHS in a patient with AS and discuss the possible mechanism underlying the type of SAHS.

**Patient concerns:**

A 46-year-old man presented with a 15-year history of AS. He had been receiving sulfasalazine for symptomatic relief and had never been on immunosuppressive therapy.

**Diagnosis:**

The patient was diagnosed with SAHS in addition to AS.

**Interventions:**

We instituted treatment with methylprednisolone (5 mg, oral, daily), leflumomide (20 mg, oral, daily), bicyclol tablets (25 mg, oral, 3 times a day), and ursodeoxycholic acid tablets (10 mg/kg, oral, daily). The patient received etanercept (50 mg, sc, once a week) as his condition deteriorated. In addition, for management of SAHS symptoms, the patient received nasal continuous positive airway pressure (CPAP) during sleep.

**Outcomes:**

Six months after commencement of the treatment, the clinical manifestations of SAHS and AS had significantly improved.

**Conclusions:**

We hypothesize that patients with AS are prone to sleep apnea due to airway compression, central depression of respiration, abnormal inflammatory responses. Hence, careful assessment toward potential SAHS symptoms should be considered especially in patients with AS.

## 1 Introduction

Ankylosing spondylitis (AS) is a multifactorial chronic inflammatory disease that predominantly affects the spine and sacroiliac joint, with a prevalence of 0.5% to 1% within the population. AS causes a variety of clinical symptoms including pain, stiffness, fatigue, physical limitations, and disturbance of sleep, all of which severely impact patients’ quality of life.^[1]^ In fact, it has been reported that the prevalence of sleep disturbances ranges from 64.8%^[2]^ to 91%^[3]^ among the patients with AS.

Sleep apnea–hypopnea syndrome is characterized by recurrent hypopnea or respiratory interruption during sleep, which can lead to excessive daytime sleepiness. Some cross-sectional studies reported that patients with certain autoimmune diseases could be predisposed to the development of SAHS through several mechanisms including: restriction of the oropharyngeal airway from temporomandibular joint involvement, or cervical spine disease causing pharyngeal and tracheal compression; cervical spine disease causing compression of the respiratory centers in the medulla, resulting in central depression of respiration; or restrictive pulmonary disease.^[4]^ Solak et al^[5]^ reported that the prevalence of SAHS in patients with AS above 35 years of age (40%) is significantly higher than in those below this age (6.3%). Furthermore, the prevalence of SAHS in patients with an AS disease duration of 5 years or longer was reported to be 3 times higher as compared with patients of a shorter disease duration. Erb et al^[4]^ suggested that SAHS could be a contributing factor to fatigue in AS, and detection and treatment of SAHS could lead to improvement of this symptom in these patients.

Therefore, in the present study, we have posed the question whether AS may also cause tracheal compression, central depression of respiration, abnormal inflammatory responses and eventually lead to the development of sleep apnea–hypopnea syndrome (SAHS). Our search of the main databases including PubMed, Elsevier, Cochrane, and the Chinese National Knowledge Infrastructure revealed AS associated with sleep apnea has been studied, but the type of sleep apnea caused by AS has been inconclusive. Previous studies have shown that most types of sleep disorders caused by autoimmune diseases are obstructive.^[4]^ But in this case, the patient developed central sleep apnea due to AS. Given this rarity we describe such a case in the present report.

## 2 Case presentation

A 46-year-old man, who had nocturnal snoring and apnea for 10 years, was admitted for further examination of his respiratory disturbances. For the past 10 years, he had been feeling a gradual increase in nocturnal snoring, fatigue, daytime sleepiness, and poor quality of sleep. His wife had noticed his occasional apnea during sleep. Additionally, the patient had a 15-year history of AS, usually with pain and morning stiffness in his lower back, and these symptoms were aggravated during rest and could be alleviated by physical activity. He did not receive standard therapy.

Physical examination revealed systolic/diastolic blood pressure of 138/80 mm Hg, pulse rate of 90/min, respiratory rate of 22 breaths/min, body temperature of 36.5°C, and body mass index (BMI) of 21.2 kg/m^2^. Examination of the respiratory system showed that breath sounds were heard bilaterally, without crackles or wheeze, and thoracic mobility was 2.5 cm. During neurological assessment we found the distal upper limb on the left muscle strength of grade 4, Babinski sign of left side (+), Chaddock sign of both side (+) and the left upper limb algesthesis weakening. We did not observe any cardiovascular or abdominal abnormalities.

Results of arterial blood gas analysis were as follows: pH 7.39, pCO_2_ 46 mm Hg, pO_2_ 48 mm Hg, HCO_3_^-^ 27.8 mmol/L, SaO_2_ 83% on room air. The results of the pulmonary function test were as follows: forced expiratory volume in 1 s (FEV_1_) of 2.64 L, predicted FEV_1_ percentage of 75%, forced vital capacity (FVC) of 2.98 L, FVC percentage predicted of 68.4%, vital capacity (VC) of 3.21 L, predicted VC percentage of 71%, FEV_1_/FVC of 106.4%, and RV/TLC of 115.1%. Laboratory results were as follows: red blood cell count of 7.39 × 10^12^/L, hemoglobin of 224 g/L, hematocrit of 66.30%, and human leukocyte antigen B27 (HLA-B27) of 89.6 U/mL. Anti-neutrophil cytoplasmic antibodies, anti-nuclear antibodies, anti-keratin antibodies, anti-phospholipid antibodies, and rheumatoid factor were all negative. Laryngoscopy examination was normal and ruled out the obstruction of larynx and pharynx. Echocardiography showed a dilated right atrium (43 mm) and right ventricle (42 mm). The velocity of the tricuspid regurgitant jet was 4.5 m/s with a pulmonary artery systolic pressure (PASP) of 51 mm Hg, and a left ventricular ejection fraction of 75%. Echocardiography showed that left ventricular diastolic function was decreased.

**Figure 1:**
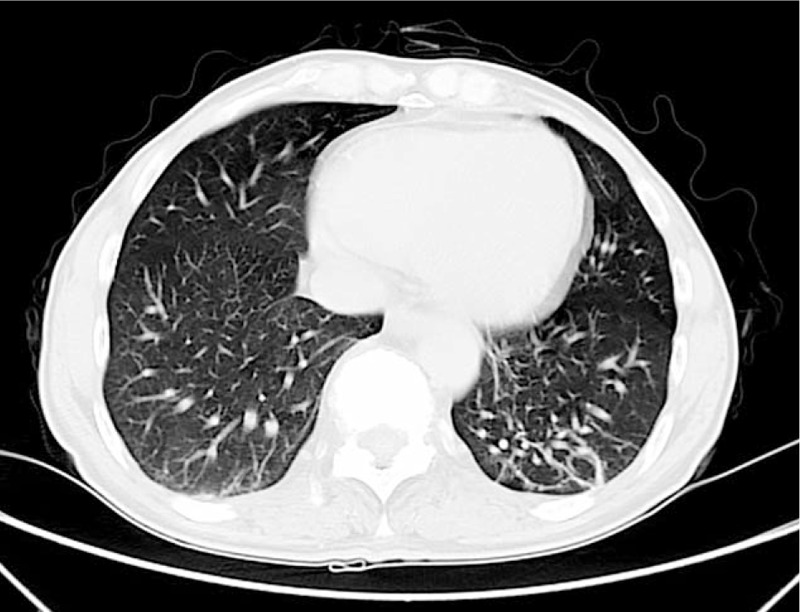
Chest CT scan revealed fibrosis within the irregular strip located in dorsal anasal segment of lower lobes.

Polysomnography (PSG) was performed and recorded parameters of electroencephalogram, chin electromyogram, oral-nasal air flow, and electrocardiogram. Based on these examinations we diagnosed mixed SAHS with an apnea–hypopneas index (AHI) of 31.5/h, oxygen desaturation index of 30.3/h and sleep efficiency of 47.7%. Mean nocturnal SaO_2_ was 80% with a minimal nocturnal SaO_2_ of 49%. There were 145 apneas and hypopneas/hour of sleep (23.4% central, 47.6% mixed, 4.8% obstructive, 24.1% hypopneas). Non-rapid eye movement (NREM) stages 1 and 2 sleep were increased to 26.3% and 59.2% of the total sleep time, respectively. NREM stage 3 sleep (4.3%) and rapid eye movement (REM) sleep (10.1%) were decreased. The number of micro-arousals was 73/h, 23 of which were related to respiratory events and 48 of which were related to spontaneity. The above data revealed a severe mixed combination of sleep apnea and central sleep apnea, which are likely to be responsible for the sleep-associated symptoms of the patient (Fig. 1).

**Figure 2:**
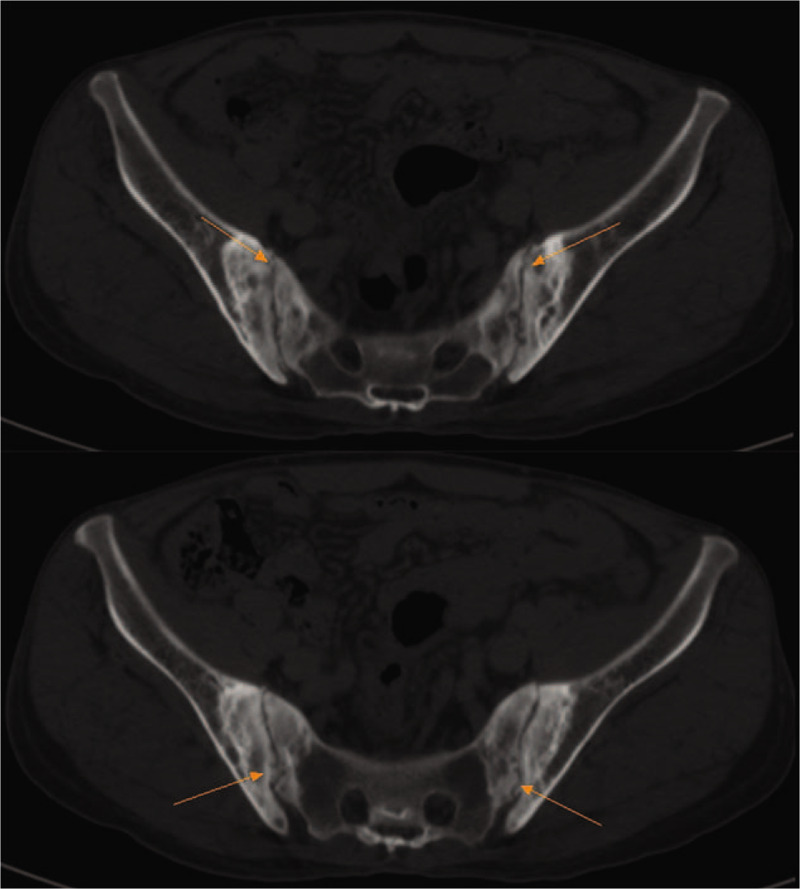
Sacroiliac joint CT revealed narrowing of bilateral sacroiliac joints space and serrated margin, multiple cystic destruction, hyperosteogeny and sclerosis of articular surface.

**Figure 3:**
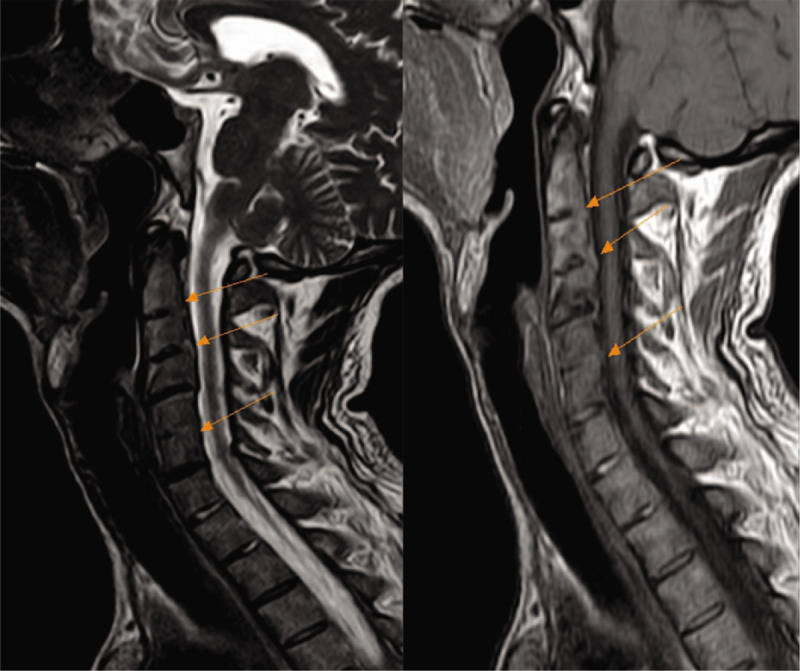
Spine magnetic resonance imaging (MRI) showed that the whole spine curvature became straight and changes of double sacroiliac joints were as follows. The changes include fusion of 2/3, 5/6 intervertebral space and mild posterior herniation of cervical 4/5, 6/7 intervertebral discs.

In addition, a contrast-enhanced chest computed tomography (CT) scan revealed fibrosis within the irregular strip located in dorsal and basal segment of lower lobes (Fig. 1). X-radiography of the sacroiliac joints supported the diagnosis of AS (Fig. 2). Spine magnetic resonance imaging (MRI) showed that the entire spine curvature had straightened and part of the intervertebral discs had integrated with each other (Fig. 3).

We instituted treatment with methylprednisolone (5 mg, oral, daily), leflumomide (20 mg, oral, daily), bicyclol tablets (25 mg, oral, 3 times a day), and ursodeoxycholic acid tablets (10 mg/kg, oral, daily). The patient received etanercept (50 mg, sc, once a week) as his condition deteriorated. Meanwhile, for management of the SAHS symptoms, the patient received nasal continuous positive airway pressure (CPAP) during sleep. Six months following commencement of the treatment, the symptoms of pain, stiffness, and sleep disturbances were largely relieved. Follow-up laboratory analysis showed a hemoglobin level of 191 g/L, red blood cell count of 5.88 × 10^12^/L, and hematocrit of 56.5%, and human leukocyte antigen B27 (HLA-B27) of 59.3 U/mL. Other biochemical tests showed: erythrocyte sedimentation rate (ESR) 12 mm/h, C reactive protein (CRP) 3.8 mg/L, which were significantly decreased compared with the initial results at the time of diagnosis. Results of arterial blood gas analysis were as follows: pH 7.35, pCO_2_ 34 mm Hg, pO_2_ 66 mm Hg, HCO_3_^−^ 23.7 mmol/L, SaO_2_ 91% on room air. The results of the PSG were as follows: apnea–hypopneas index (AHI) of 21.4/h, oxygen desaturation index of 17.5/h and sleep efficiency of 68.1%. Mean nocturnal SaO_2_ was 87% with a minimal nocturnal SaO_2_ of 62%. In addition, the patient was discharged and followed-up one and a half years, the symptoms of dyspnea, pain, stiffness, and sleep disturbances were alleviated.

## 3 Discussion

Ankylosing spondylitis is a chronic, progressive, inflammatory rheumatic disease that affects the axial skeleton and peripheral joints, causing characteristic inflammatory back pain, which can lead to structural and functional impairments as well as a decrease in quality of life. This disease predominantly affects young people, who generally present at around 26 years of age.^[6]^ Young age at onset of symptoms is associated with worse functional outcomes.^[7]^

Patients with AS have significant problems arising from pain, stiffness, fatigue, and sleep disturbances. Various sleep-associated problems are common including poor quality of sleep, sleep onset insomnia, difficulty awakening, and obstructive sleep apnea syndrome.^[8]^ Abdulaziez et al^[9]^ have shown that patients with AS have a higher percentage of light sleep (stages I and II), a lower percentage of deep, slow wave sleep (SWS), and decreased sleep efficiency compared with healthy control subjects. A case–control study has demonstrated that sleep disturbances are associated with increased pain, disease activity, depression, and anxiety in patients with AS.^[10]^ Furthermore, recent evidence has suggested a possible role for restless legs syndrome (RLS) in the regulation of sleep, providing further reason for exploring sleep patterns in patients with inflammatory rheumatic disease.^[11]^ Weinstick et al^[12]^ have observed that a high incidence of RLS has been found previously in patients with AS, and that uncomfortable leg sensations may be an important aspect of poor sleep within AS. Patients with AS reported priority for improvement in sleep disturbances significantly more frequently than other inflammatory arthropathies. These data further encourage the assessment of sleep-associated problems in AS.^[13]^

Sleep apnea–hypopnea syndrome is characterized by recurrent hypopnea or respiratory interruption during sleep, which causes intermittent hypoxemia, hypercapnia, and sleep structure disturbances. It has been reported that patients with certain autoimmune diseases may be predisposed to developing SAHS through several mechanisms including restriction of the oropharyngeal airway from temporomandibular joint involvement, cervical spine disease causing pharyngeal and tracheal compression or compression of the respiratory centers in the medulla, as well as restrictive pulmonary disease.^[4]^ It is known that age is a major factor affecting the prevalence of SAHS in patients with AS, whereby being older than 35 years of age raises the risk of developing SAHS from 6.3% to 40%.^[5]^ Furthermore, the prevalence of SAHS in patients with AS with a disease duration of more than 5 years increases the risk compared to those with a shorter disease duration by 3-fold. Observations by Erb et al^[4]^ indicate that SAHS may be a contributing factor to fatigue in AS, and thus detection and treatment of SAHS may alleviate such symptoms in these patients.

However, Kang et al^[14]^ reported that the overall prevalence rate for autoimmune disease was 1.88 times greater in patients with SAHS than in the non-SAHS patients. Several possible explanations for this study including: First, the potential link between SAHS and the development of autoimmune disease may be associated with the abnormal inflammatory status induced by SAHS. A finding worth noting is that elevated tumor necrosis factor α(TNF-α) and IL-6 levels in SAHS patients have been observed and correlated with daytime sleepiness and disease severity.^[15]^ Meanwhile, RA, AS, and inflammatory bowel disease are reported associated with dysregulation of TNF-α signaling.^[16]^ Second, SAHS and autoimmune disease may share some genetic background and risk factors. For instance, obesity is a prevalent problem among patients with SAHS. Adipose tissue can releases several adipokines, which can act as regulatory chemokines in the immune system and contribute to autoimmunitiy.^[17]^ Third, the common use drug treatments in AS are Nonsteroidal anti-inflammatory drugs (NSAID) and sulfasalazine. Their side effect is to result in puffiness of face which indirectly decreases the upper airway space. That is, it is a chicken-and-egg problem to determine the direction of causation between SAHS and AS patients.

In the present case, the patient's types of sleep dyspnea were central and mixed. We considered the causes of central sleep dyspnea in patients, including several mechanisms. First, during the progression of AS, MRI suggests that the fusion of high cervical vertebra may directly compress the respiratory center.^[18,19]^ Second, as demonstrated by previous studies, pulmonary stroma changes occur in autoimmune disease at the advanced stage, which may have aggravated anoxia symptoms of the patient. Nocturnal intermittent hypoxia and hypercapnia can damage the respiratory center and reduce the responsiveness of the respiratory center.^[5]^ Third, the patient's echocardiography indicates that enlargement of the right heart and pulmonary hypertension, which is also a high risk factor for central sleep dyspnea.^[20]^

Meanwhile, the patient had a 15-year history of AS, during which he had not received standard treatment. Consequently, as described above, systemic inflammation and immune dysfunction may result in the development of OSAHS. Additionally, during the progression of AS, ribs and cervical vertebrae have been invaded. This may cause a compression of the airway and affect the expansion of the chest, which might result in limited thoracic expansion capacity and significantly decreased expansion ratio, leading to low oxygen saturation. Being subjected to long-term compression, limited chest activity, and nocturnal sleep apnea, the patient may therefore have been in a state of hypoxia for a long time, leading to a secondary increase in the number of erythrocytes. All of the above reasons make patients prone to obstruction sleep dyspnea. Thus, the association of AS and SAHS may not be a mere coincidence but could be linked by several pathomechanisms, with potential diagnostic implications for patients with both AS and SAHS.

The basis of treating AS includes a combination of physical exercise and pharmacological treatments. NSAIDS are a first-line therapy, and TNF-α inhibitors are a second-line medication for patients with persistently high disease activity despite conventional pharmacological treatment.^[21]^ Erdal et al^[22]^ found that using anti-TNF therapy could decrease not only sleep disturbances but also daytime fatigue and the frequency of headaches after waking up. AS patients with SAHS and AHI > 15 were treated with nasal continuous positive airway pressure (CPAP) at home, while AHI < 15 were recommended to lose weight and avoid cigarette and alcohol. Careful assessment toward potential SAHS symptoms should be considered especially in patients with AS older than 35 years of age, and with disease duration of more than 5 years. Taking this into account will enhance the chances of a timely diagnosis and therefore early and proper treatment, which can significantly improve patients’ symptoms and quality of life.

## Author contributions

All authors diagnosed this disease and collected data, Yan Wang and Shan Lin wrote the draft of this article, Wei Guan revised this article. Yan Wang and Shan Lin contributed equally to this work. Informed written consent was obtained from the patient for publication of this case report and accompanying images.

Data curation: Chenxi Li, Yingqing Shi.

Writing: original draft: Yan Wang, Shan Lin.

Writing: review & editing: Wei Guan.
